# Fabrication of hyaline-like cartilage constructs using mesenchymal stem cell sheets

**DOI:** 10.1038/s41598-020-77842-0

**Published:** 2020-11-30

**Authors:** Hallie Thorp, Kyungsook Kim, Makoto Kondo, David W. Grainger, Teruo Okano

**Affiliations:** 1 Department of Pharmaceutics and Pharmaceutical Chemistry, Cell Sheet Tissue Engineering Center (CSTEC), University of Utah, 30 South 2000 East, Salt Lake City, UT 84112 USA; 2 Department of Biomedical Engineering, University of Utah, Salt Lake City, UT USA; 3 Institute of Advanced Biomedical Engineering and Science, Tokyo Women’s Medical University, Tokyo, Japan

**Keywords:** Mesenchymal stem cells, Stem-cell differentiation, Cytoskeleton, Cartilage, Biomaterials - cells, Tissue engineering

## Abstract

Cell and tissue engineering approaches for articular cartilage regeneration increasingly focus on mesenchymal stem cells (MSCs) as allogeneic cell sources, based on availability and innate chondrogenic potential. Many MSCs exhibit chondrogenic potential as three-dimensional (3D) cultures (i.e. pellets and seeded biomaterial scaffolds) in vitro; however, these constructs present engraftment, biocompatibility, and cell functionality limitations in vivo. Cell sheet technology maintains cell functionality as scaffold-free constructs while enabling direct cell transplantation from in vitro culture to targeted sites in vivo. The present study aims to develop transplantable hyaline-like cartilage constructs by stimulating MSC chondrogenic differentiation as cell sheets. To achieve this goal, 3D MSC sheets are prepared, exploiting spontaneous post-detachment cell sheet contraction, and chondrogenically induced. Results support 3D MSC sheets’ chondrogenic differentiation to hyaline cartilage in vitro via post-contraction cytoskeletal reorganization and structural transformations. These 3D cell sheets’ initial thickness and cellular densities may also modulate MSC-derived chondrocyte hypertrophy in vitro. Furthermore, chondrogenically differentiated cell sheets adhere directly to cartilage surfaces via retention of adhesion molecules while maintaining the cell sheets’ characteristics. Together, these data support the utility of cell sheet technology for fabricating scaffold-free, hyaline-like cartilage constructs from MSCs for future transplantable articular cartilage regeneration therapies.

## Introduction

Articular cartilage defects are increasingly responsible for morbidity and compromised quality of life in the global population^1^. Lacking a direct blood supply, articular cartilage has minimal ability to repair spontaneously; therefore, cartilage injuries are rarely able to heal without intervention, and often progress to osteoarthritis (OA)^1–3^. Healthy articular cartilage exhibits hyaline structure and characteristics; therefore, the goal for cartilage regeneration therapies is promotion of these hyaline-like phenotypes at the site of injury^1,4,5^. Bone marrow stimulation techniques, such as microfracture, are the most frequently used method in clinical practice^6^; however, the resulting mixed hyaline/fibrocartilage tissue is inferior to native hyaline cartilage, specifically in its ability to withstand compressive forces, diminishing functionality of the regenerated cartilage in vivo^6,7^. Improved treatment options that quickly and reliably regenerate hyaline cartilage must be developed to properly treat articular cartilage focal defects before they progress to OA.

Diverse cell therapies have been developed for attempted treatment of articular cartilage focal defects^6^. Many of these therapies employ autologous chondrocyte sourcing (i.e. patient is both the cell donor and recipient) which presents limitations of patient burden of multiple surgeries, donor-dependent cell quality, and costly, lengthy cell expansion and preparation times^6,8^. Growing efforts to transition from autologous to allogeneic cell sources to prepare readily-available cell therapies increasingly advocate use of mesenchymal stem cells (MSC) as an allogeneic cell source^9–11^. MSCs offer well-documented regenerative properties, standards for preparing cells with specific phenotypes, and multipotency, including chondrogenic lineages^12–15^. Even though many reports demonstrated that undifferentiated MSC therapies show some therapeutic efficacy in cartilage regeneration, their differentiation fate is still not easily controlled in vivo, resulting in mixed hyaline/fibrocartilage tissue similar to that seen from microfracture and extended time in vivo for any regenerative therapeutic effect^16–19^. Therefore, next-generation approaches focus on using MSCs to create hyaline cartilage constructs in vitro that will be able to more rapidly and reliably replace damaged articular cartilage.

In vitro MSC chondrogenic differentiation within 3D constructs (e.g. pellet cultures) is well-established^14,20–22^; however, for in vivo cell delivery, these cell cultures are usually dissociated enzymatically and delivered as cell suspensions or seeded into scaffold materials for implantation^6,10,23,24^, resulting in poor localization to the target tissue site and poor retention of hyaline-like phenotypes at time of transplantation^25,26^. Pellet cultures specifically are limited by adhesion and homogeneity constraints^27,28^. Extensive work has been reported for tailoring scaffold materials, such as collagens, alginates, hyaluronic acid, or PGA/PLA, to accommodate cells and promote more homogenous chondrogenic differentiation^23,29,30^. However, even when these cell-seeded scaffolds achieve in vitro chondrogenic differentiation, the presence of scaffold materials or necessary exogenous adhesives inhibit direct communication between transplanted cells and the target tissue, resulting in poor functional regeneration post-implantation^9,11,23^. Current advances in scaffold-free 3D differentiation have prompted more homogenous chondrogenic differentiation in more ergonomic disc shapes without the limitations of scaffold materials^31–35^. However, these scaffold-free disc-shaped constructs rely on standard culture plasticware and exorbitant seeding densities, requiring mechanical detachment post-differentiation, damaging the cells and reducing adhesion capabilities for transplantation^31,33,34,36,37^. Despite promising advances in tissue engineering and many potential advantages of MSCs for cartilage regeneration, no available methods for preparing MSC-derived chondrogenic constructs reliably retain chondrogenic potential in vitro and allow direct, unassisted in vivo transplantation of chondrogenically differentiated hyaline-like constructs capable of interfacing with host tissue^23,38,39^.

Cell sheet tissue engineering, using temperature-responsive cultureware, produces scaffold-free, 3D cell sheet constructs^40–42^. Regenerative cells are harvested as intact sheets with reproducible physiologic properties and scalable production methods^43–45^. Cell sheets retain endogenous extracellular matrix (ECM), receptors, and adhesive proteins, enhancing cell viability and communication and permitting spontaneous adhesion to biomaterials and biologic surfaces without sutures or glues^40,44^. Cell sheet technology has shown preliminary success for articular cartilage regeneration using autologous and allogeneic chondrocytes^46–49^. However, while chondrocyte sheets in those studies exhibit some ability to induce hyaline phenotypes in vivo, they do not inherently exhibit these phenotypes prior to knee implantation due to de-differentiation of chondrocytes during cell culture in vitro and are currently supported by microfracture surgery to supply endogenous progenitor cells^47^. Therefore, hyaline tissue formation in vivo post-transplantation requires extended time, hindering direct and rapid replacement of damaged articular cartilage. As a next-generation approach, this study represents a “back-to-bench” strategy to further build upon established cell sheet precedent and support development of future cartilage regeneration therapies.

**Figure 1:**
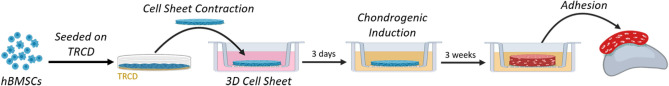
In vitro engineering of adhesive, scaffold-free, 3D hyaline-like cartilage tissue from human bone marrow-derived mesenchymal stem cell (hBMSC) sheets. Our approach uses cell sheet tissue engineering to prepare scaffold-free 3D MSC cellular constructs via spontaneous post-detachment cell sheet contraction from temperature-responsive culture dishes (TRCD). This technology allows in vitro differentiation to hyaline-like cartilage phenotypes by re-plating 3D contracted sheets to cell culture inserts and inducing with chondrogenic media for 3 weeks. Direct adhesion of sheets to target tissue post-differentiation is also possible without damaging the structure or chondrogenic characteristics of the sheet construct. (Created with BioRender.com).

This study fabricates ready-to-use, hyaline-like cartilage constructs from MSCs in vitro using cell sheet technology. Data further demonstrate that cell sheets retain adhesion capability after chondrogenic differentiation to hyaline-like phenotypes, allowing spontaneous attachment without damaging the chondrogenic construct (Fig. 1). This study lays the foundation for developing a pre-differentiated hyaline-like cell sheet construct that will be able to reduce the time for transplanted cells to establish hyaline cartilage in vivo for future regenerative therapies.

## Results

### Spontaneous post-detachment contraction of hBMSC sheets

**Figure 2:**
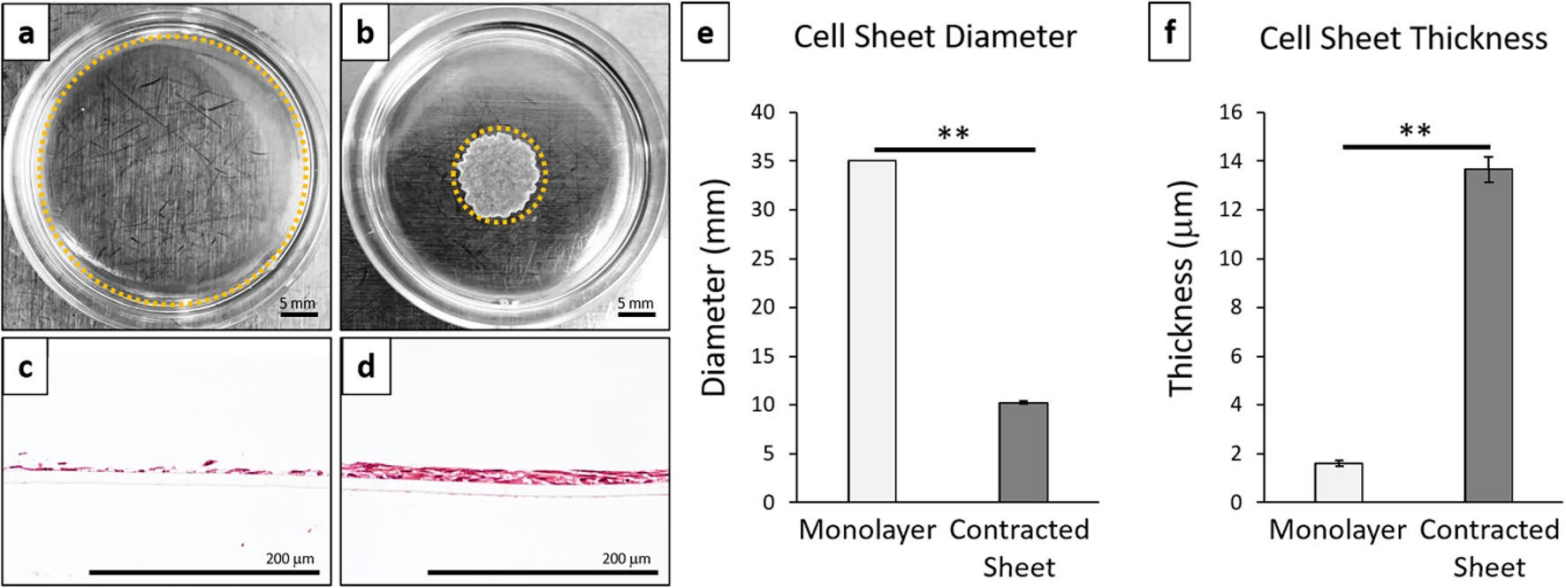
Cell sheet contraction promotes 3D structural rearrangements. Representative macroscopic images of cell culture constructs: (**a**) 2D monolayer cultures, (**b**) 3D contracted cell sheets. Cell construct edges marked by dotted orange line. Representative cross-sectional histology of H&E for (**c**) 2D monolayer culture and (**d**) 3D contracted cell sheet. Cell construct (**e**) diameter and (**f**) thickness for monolayer cultures compared to contracted cell sheets. Error bars represent means ± SD (*n* = 4) (**p < 0.01). Microscopic photos acquired with AmScope Software (v4.8.15934, https://www.amscope.com/software-download) (**c**,**d**).

Cultured human bone marrow-derived mesenchymal stem cell (hBMSC) sheets spontaneously contracted after detachment from temperature-responsive culture dishes (Fig. 2). Spontaneous post-detachment contraction considerably altered the size and structure of the resulting 3D contracted cell sheet (Fig. 2b,d) compared to the 2D monolayer cell culture conditions (Fig. 2a,c). hBMSC sheets showed significant reductions in diameter (3.5-fold decrease, *p* = 1.77E−5) (Fig. 2e) and increased thickness (sevenfold increase, *p* = 3.63E−6) (Fig. 2f) after post-detachment cell sheet contraction compared to 2D monolayer cell culture conditions.

### Sheet cytoskeletal changes for chondrogenic potential

**Figure 3:**
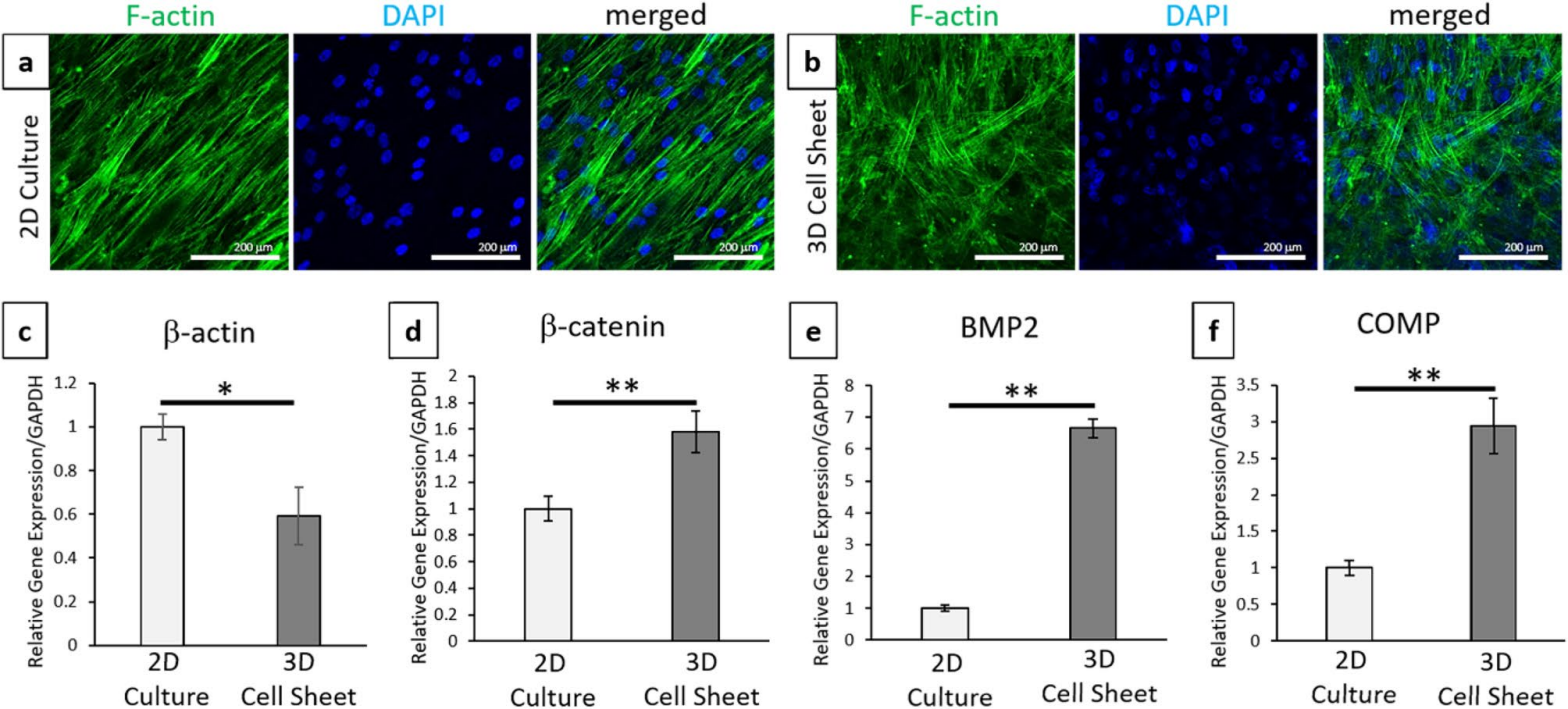
Cell sheet contraction promotes cytoskeletal rearrangements and pro-chondrogenic signaling compared to 2D cell culture. Representative confocal images of full dish (top–down) (**a**) 2D cultures and (**b**) 3D contracted sheets before chondrogenic induction at day-0 with phalloidin/F-actin (green) staining for cytoskeleton and DAPI (blue) nuclear staining. Quantitative real-time PCR gene expression for (**c**) cytoskeletal structure marker β-actin, (**d**) cell–cell marker β-catenin, and chondrogenic ECM markers (**d**) BMP2 and (**f**) COMP. All gene expression normalized to GAPDH and compared to the 2D cell culture 0-day control sample. Error bars represent means ± SD (*n* = 4) (*p < 0.05, **p < 0.01). Microscopic photos acquired with NIS Elements AR Software (v.4.30.01, https://www.nikon.com/products/microscope-solutions/support/download/software/imgsfw/nis-ar_v43001du164.htm) (**a**,**b**).

Cultured hBMSC sheet cytoskeletal arrangements were observed with phalloidin (F-actin) fluorescent staining, and nuclei were identified by DAPI (Fig. 3a,b). At day 0, cells within 2D cultures exhibited elongated and aligned cytoskeletal structures associated with standard adherent culture of MSCs (Fig. 3a). Conversely, harvested hBMSC cell sheets allowed to contract after detachment exhibited a more random, crossed-fiber structure with more rounded nuclei (Fig. 3b). These cytoskeletal changes were confirmed as significant based on relative β-actin gene expression (Fig. 3c) between contracted 3D sheets and 2D monolayer cultures (*p* = 0.0194). In addition to these cytoskeletal changes, contracted 3D sheets showed significant increases in cell–cell interactions, β-catenin (*p* = 0.00865), and pro-chondrogenic signaling molecules, BMP2 (*p* = 0.0000457) and COMP (*p* = 0.000947), (Fig. 3d–f) compared to 2D monolayer cultures prior to chondrogenic induction.

### Increased chondrogenic potential of hBMSCs as 3D contracted sheets

**Figure 4:**
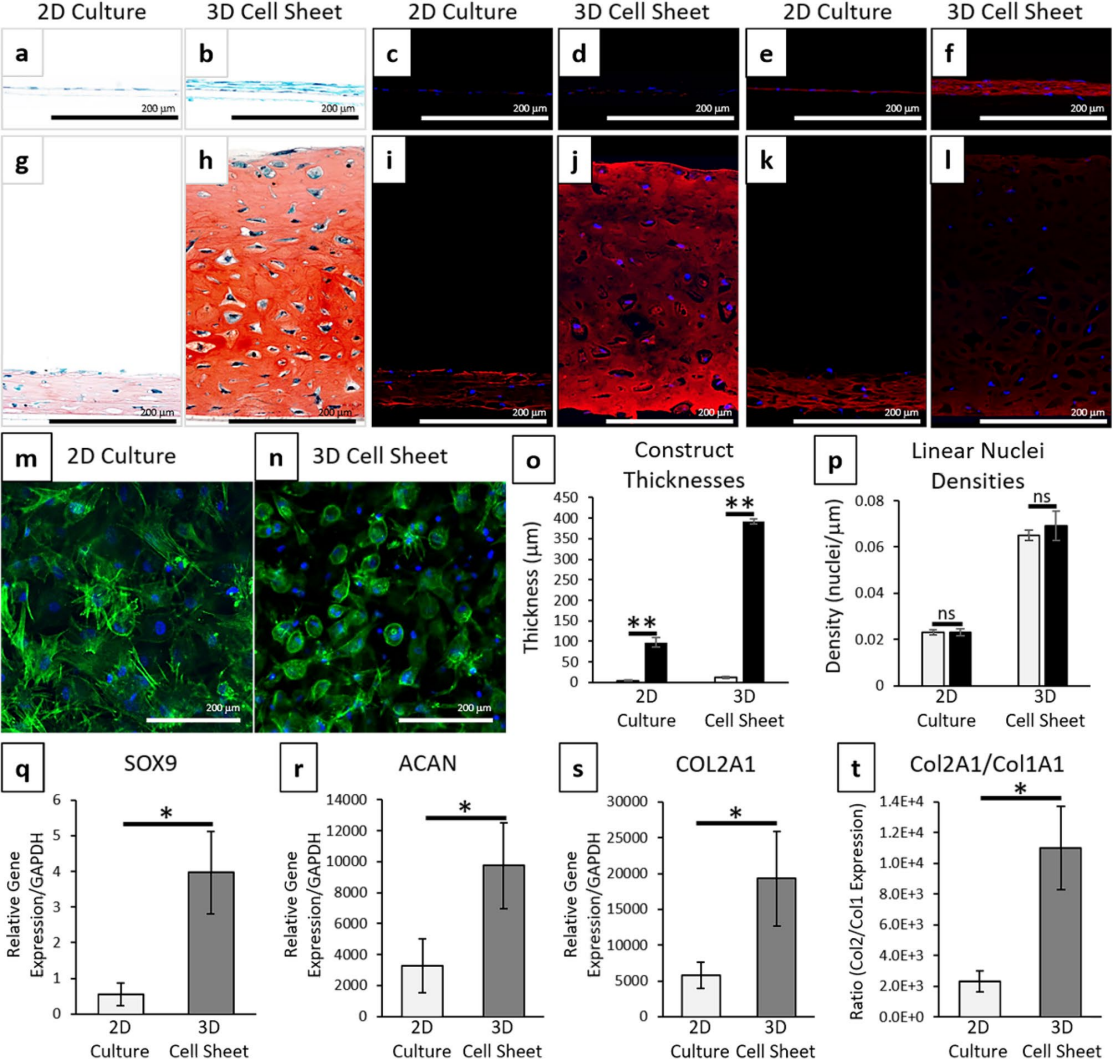
Cell sheet contraction stimulates enhanced chondrogenic differentiation of hBMSCs. Comparison between 3-week chondrogenically induced hBMSC sheets before (2D culture) and after (3D cell sheet) post-detachment sheet contraction. Representative images of histological cross-sections of hBMSC constructs in (**a**–**f**) control medium and (**g**–**l**) chondrogenic medium for 3 weeks. Stains were (**a**,**b**,**g**,**h**) Safranin-O/Fast green for sulfated proteoglycans, (**c**,**d**,**i**,**j**) type II collagen (pseudo red) with DAPI (blue), and (**e**,**f**,**k**,**l**) type I collagen (red) with DAPI (blue). Representative full dish (top-down) confocal images of 3-week chondrogenically-induced (**m**) 2D cultures and (**n**) 3D cell sheets with phalloidin/F-actin (green) staining for cytoskeleton and DAPI (blue) nuclear staining. Cell sheet (**o**) thicknesses and (**p**) linear nuclei density for 2D cultures and 3D cell sheets at 0-day (grey bar) and 3-week (black bar) differentiation. Chondrogenic gene expression with quantitative real-time PCR for (**q**) SOX9, (**r**) aggrecan, and (**s**) type II collagen. (**t**) Ratios of type II to type I collagen shown as ratios of relative gene expression. All gene expression normalized to GAPDH and compared to the 2D culture 3-week control samples. Error bars represent means ± SD (*n* = 4) (ns: *p* ≥ 0.05, *p < 0.05, **p < 0.01). Microscopic photos acquired with AmScope Software (v4.8.15934, https://www.amscope.com/software-download) (**a**,**b**,**g**,**h**), Zeiss Zen software (v.2.7, https://www.zeiss.com/microscopy/us/products/microscope-software/zen.html) (**c**–**f**, **i**–**l**), and NIS Elements AR Software (v.4.30.01, https://www.nikon.com/products/microscope-solutions/support/download/software/imgsfw/nis-ar_v43001du164.htm) (**m**,**n**).

hBMSCs were chosen as an MSC source with documented chondrogenic potential^14^, which was confirmed in standard 3D pellet cultures (see Supplementary Fig. S1). 3-week chondrogenic differentiation of hBMSCs harvested as 3D contracted cell sheets resulted in positive hyaline-like chondrogenesis (Fig. 4). Positive Safranin-O and type II collagen staining were identified in 3-week differentiated samples compared to control samples (Fig. 4a–d,g–j). Safranin-O stains sulfated proteoglycans red (depth of red color is relative to GAG content) with Fast Green counterstaining other ECM proteins blue. Type II collagen is denoted by red fluorescence (pseudo red immunostaining) and nuclei are counterstained with DAPI (blue). Monolayer 2D hBMSC cultures exhibited some slightly positive chondrogenic staining with Safranin-O (Fig. 4g) and type II collagen (Fig. 4i) after 3-week differentiation. The 3D contracted sheets after 3-week differentiation stained more intensely for all chondrogenic markers [Safranin-O (Fig. 4h) and type II collagen (Fig. 4j)] compared to the chondrogenic 2D cultures (Fig. 4g,i). The differentiated 3D contracted sheets also developed lacunae structures associated with mature hyaline cartilage (Fig. 4h)^50^. After 3 weeks of differentiation, most cells in the 3-week differentiated contracted sheets exhibited condensed, rounded cytoskeletal structures associated with mature chondrocytes (Fig. 4n)^26,51^. Some cells in 2D monolayer cultures displayed this rounded cytoskeletal structure; however, the majority of cells in the 3-week differentiated monolayers had more variable spindle-like or fibroblastic cell shapes (Fig. 4m). Gene expression of all chondrogenic markers were significantly increased (SOX9 (Fig. 4q) (*p* = 0.0144), ACAN (Fig. 4r) (*p* = 0.0136), and COL2A1 (Fig. 4s) (*p* = 0.035)) in 3D contracted cell sheets compared to 2D cell cultures after 3 weeks of differentiation. 3D contracted cell sheets also expressed significantly higher ratios of type II to type I collagen (Fig. 4t) (*p* = 0.0108) with minimal staining for type I collagen (Fig. 4l) compared to 2D cultures (Fig. 4k) after 3 weeks of differentiation. Additionally, 3D contracted sheets showed a 30-fold increase in thickness after 3 weeks of differentiation, significantly greater than the 23-fold increase in 2D cell culture thickness (Fig. 4o) (*p* = 4.26E−6). This increase in thickness after differentiation did not significantly change the number of cells within either the 3D contracted cell sheets or 2D cell cultures (Fig. 4p) [*p* = 0.422 (3D); 0.997(2D)], suggesting that increased thickness of chondrogenic cell sheets results from chondrogenically induced ECM deposition.

### Chondrogenic differentiation of cell sheets over time

**Figure 5:**
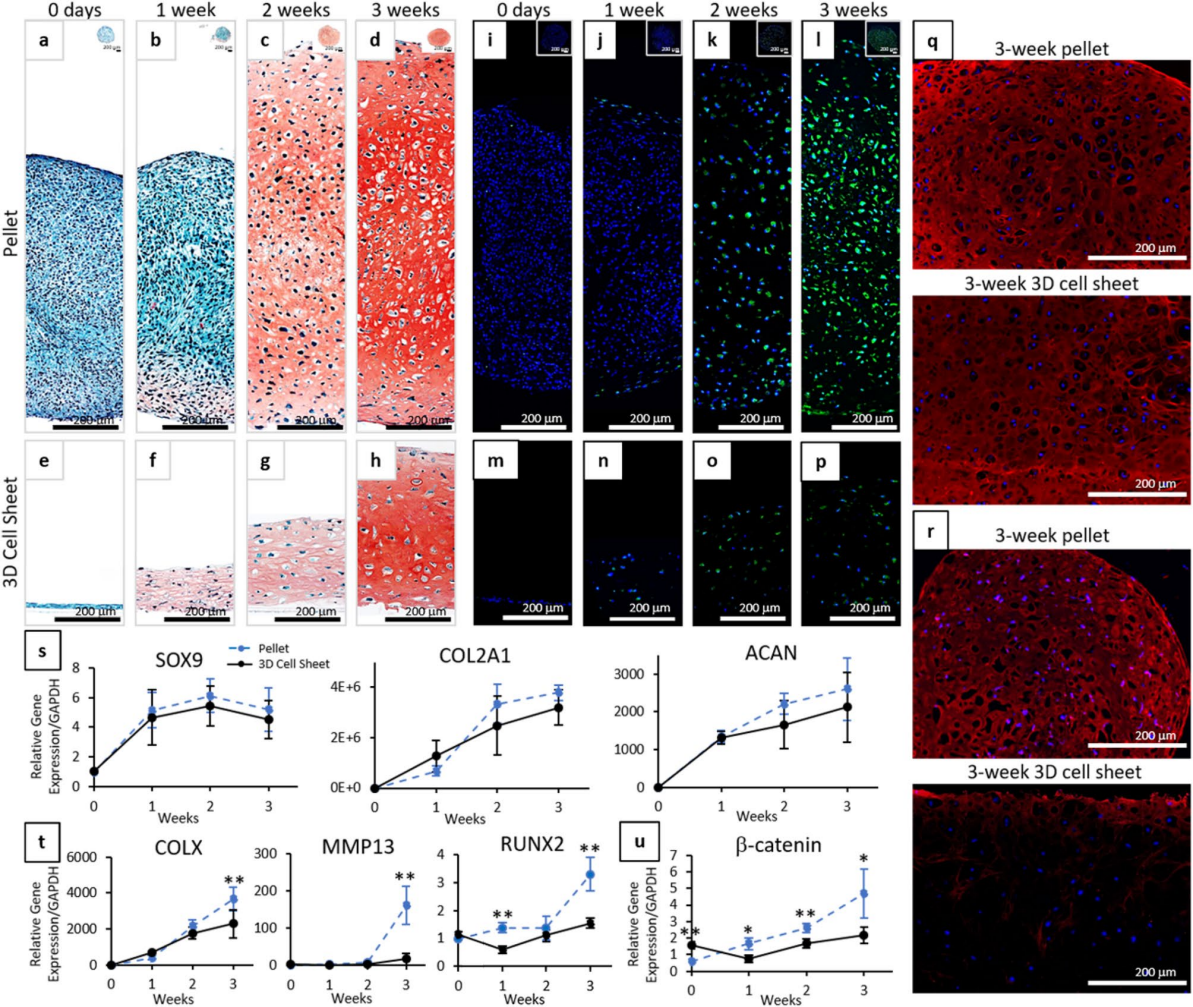
Progression of chondrogenic differentiation over time for 3D hBMSC cell sheets compared to standard pellet cultures. Representative images of histological cross-sections of (**a**–**d**, **i**–**l**) pellets and (**e**–**h, m**–**p**) 3D cell sheets in chondrogenic medium for 0 days–3 weeks stained with (**a**–**h**) Safranin-O/Fast green for sulfated proteoglycans and (**i**–**p**) MMP13 (green) with DAPI (blue). Representative images of histological cross-sections of pellets and 3D cell sheets at 3 weeks in chondrogenic media stained for (**q**) type II collagen (pseudo red) and (**r**) type I collagen (red) with DAPI (blue). Quantitative real-time PCR of pellets (dashed blue) and 3D cell sheets (solid black) for (**s**) chondrogenic gene expression: SOX9, type II collagen, and aggrecan; (**t**) hypertrophic and fibrocartilage gene expression: type X collagen, MMP13, and RUNX2; (**u**) cell–cell interaction expression: β-catenin. All gene expression normalized to GAPDH and compared to single cell 0-day control samples. Error bars represent means ± SD (*n* = 4). (*p < 0.05, **p < 0.01). Microscopic photos acquired with AmScope Software (v4.8.15934, https://www.amscope.com/software-download) (**a**–**h**) and Zeiss Zen software (v.2.7, https://www.zeiss.com/microscopy/us/products/microscope-software/zen.html) (**i**–**p**,**q**,**r**).

Chondrogenic differentiation of 3D contracted harvested cell sheets and pellets over time showed very similar progressions in chondrogenesis, with slightly earlier onset of sulfated GAG accumulation and delayed onset of hypertrophic and fibrocartilage phenotypes seen in 3D contracted hBMSC sheets (Fig. 5). Pellet cultures are a standard positive control for in vitro MSC chondrogenesis as they have been shown to successfully express chondrogenic characteristics comparable to human cartilage tissue^52^. The comparison to pellet cultures, rather than the physiologically static human cartilage tissue, is also beneficial in allowing direct comparisons of chondrogenic differentiation of MSCs over time. Both pellet cultures and 3D sheets were negative for Safranin-O staining at day-0 (Fig. 5a,e). After 1-week differentiation, 3D contracted cell sheets (Fig. 5f) showed greater Safranin-O staining throughout the sample than pellet cultures (Fig. 5b), but staining was faint for both samples. By 3-week differentiation, both 3D cell sheets (Fig. 5h) and pellet cultures (Fig. 5d) stained strongly for Safranin-O (Fig. 5h,d) and type II collagen (Fig. 5q), while exhibiting lacunae structures. Gene analysis showed a similar trend for chondrogenic marker expression (SOX9, COL2A1, ACAN) over 3-weeks of differentiation for contracted sheets and pellets (Fig. 5s). Chondrogenic marker expression was not significantly different between the 3D sheets and pellet cultures at any time point during differentiation (Sox 9: *p* = 0.2257; COL2A1: *p* = 0.3046; ACAN: *p* = 0.5389).

Although chondrogenic expressions were similar, 3D contracted sheets exhibited delayed onset of hypertrophic and fibrocartilage characteristics compared to standard pellet cultures. Immunohistochemical staining for hypertrophic marker, MMP13^53,54^, was negative in day-0 pellet cultures and 3D sheets (Fig. 5i,m). MMP13 staining in hBMSC pellet cultures remained negative or minimal through 2 weeks (Fig. 5i–k), but was highly expressed in 3-week samples (Fig. 5l). MMP13 staining remained negative or minimal in 3D sheet samples through 3 weeks (Fig. 5m–p). By 3-week differentiation, pellet cultures were stained strongly for fibrocartilage marker type I collagen, whereas 3D contracted sheets showed minimal type I collagen positive staining (Fig. 5r). Hypertrophic type X collagen^55, 56^ gene expression increased throughout chondrogenic differentiation for both 3D sheets and pellets (Fig. 5t); however, expression of type X collagen was significantly higher in pellet culture than in 3D sheets after 3-weeks of differentiation (*p* = 0.0087). MMP13 gene expression was low during early chondrogenesis for both constructs, but significantly higher in pellets compared to 3D contracted cell sheets at 3 weeks (Fig. 5t) (*p* = 0.0012). Expression of pre-osteogenic marker, RUNX2, did not increase significantly throughout the course of differentiation for 3D cell sheets (*p* = 0.159) and was significantly higher in pellet cultures compared to 3D cell sheets at 1 week (*p* = 0.000352) and 3 weeks (*p* = 0.00159). Expression of cell–cell adhesion marker β-catenin, related to chondrogenic commitment during early stages of chondrogenesis but also associated with chondrocyte hypertrophy if overexpressed during late stages of chondrogenic differentiation^57–59^, was significantly higher in 3D cell sheets at the time of induction, but then significantly lower throughout differentiation compared to hBMSC pellet cultures (Fig. 5u) (*p* = 0.0058 (all time points); *p* = 0.00248 (0 day); 0.0374 (1 W); 0.00591 (2 W); 0.0464 (3 W)).

### Hyaline-like cell sheet manipulation without affecting sheet characteristics

**Figure 6:**
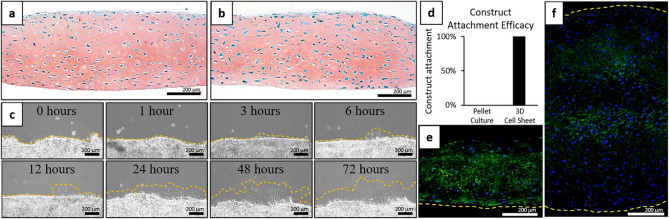
Manipulation and secondary adhesion capabilities of 3D contracted hBMSC cell sheets and pellet cultures post-differentiation to FBS coated surfaces. Representative images of Safranin-O stained histological cross-sections of 3-week differentiated 3D sheets (**a**) before transfer and (**b**) 3 days after transfer to an FBS-coated surface. (**c**) Representative phase-contrast images of sheet edges from 0 to 72 h after transfer. Cell migration from cell sheet edges marked by dotted orange line. (**d**) Attachment efficacy of 3-week differentiated pellets and 3D cell sheets after 1 h of attachment to secondary FBS-coated culture dishes and 6 h continued culture (*n* = 6). Construct attachment = ((number of attached constructs)/(number of attempted transferred constructs)) * 100. Representative IHC cross-sectional images of 3-week differentiated (**e**) contracted sheets and (**f**) pellet cultures for expression and localization of adhesion molecule laminin (green) with DAPI (blue) nuclear stain. Adhesion surfaces ((**e**) Basal side of the cell sheet and (**f**) periphery of the pellet culture) marked by dotted yellow lines. Microscopic photos acquired with AmScope Software (v4.8.15934, https://www.amscope.com/software-download) (**a**–**c**) and Zeiss Zen software (v.2.7, https://www.zeiss.com/microscopy/us/products/microscope-software/zen.html) (**e**,**f**).

3D contracted hBMSC sheets differentiated for 3 weeks were able to be manipulated and transferred as intact sheets to new culture surfaces (Fig. 6). After 3 days of secondary culture on FBS-coated surfaces, cell sheets were harvested, fixed, and stained with Safranin-O. This staining showed no discernible changes to the structure (e.g. cell sheet thickness, lacunae, cellular distribution within the ECM) or GAG composition of the cell sheets after transfer (Fig. 6a,b). During secondary culture, cell migration/proliferation was observed at edges of the cell sheets as early as 6 h after transferring the harvested, differentiated, 3D contracted sheets (Fig. 6c), indicating rapid, spontaneous surface adhesion and cell viability. Compared to 100% attachment success rate for the 3-week differentiated cell sheets (6/6 sheets adhered completely), none of the re-plated 3-week differentiated pellets were able to adhere and remain attached to the secondary culture dish (0/6 pellets adhered) after fresh media was added and constructs were cultured for an additional 6 h (Fig. 6d). Immunohistochemical analysis of the 3-week differentiated 3D contracted sheets and pellets showed that adhesion molecule laminin staining was strongly expressed along the cell sheet basal side (Fig. 6e), whereas laminin staining of the pellet cultures showed minimal positive staining around the pellets’ periphery (i.e. the interface surface) (Fig. 6f).

### Hyaline-like cell sheet adhesion capabilities

**Figure 7:**
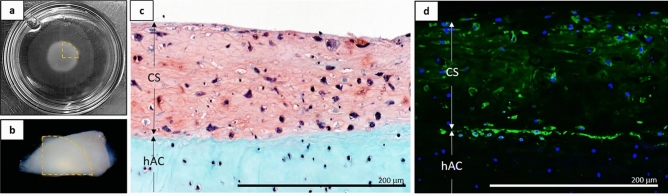
Adhesion characteristics for 3D contracted hBMSC cell sheets post-differentiation to fresh ex vivo human cartilage pieces. (**a**) Cell sheets after 3-week differentiation in 35 mm culture dish (**b**) transferred onto fresh ex vivo human articular cartilage samples. Transferred cell sheet quadrant marked by dotted orange line. Representative cross-sectional histological and IHC staining of 3-week chondrogenic 3D hBMSC sheets (CS) naturally adhered to fresh ex vivo human articular cartilage (hAC) for (**c**) Safranin-O/Fast-green and (**d**) cell adhesion molecule laminin (green) counterstained with DAPI (blue). Microscopic photos acquired with AmScope Software (v4.8.15934, https://www.amscope.com/software-download) (**c**) and Zeiss Zen software (v.2.7, https://www.zeiss.com/microscopy/us/products/microscope-software/zen.html) (**d**).

3D contracted hBMSC sheets differentiated for 3 weeks were able to spontaneously and strongly adhere to fresh, ex vivo, human articular cartilage pieces (Fig. 7). Post-differentiation 3D hBMSC sheets (Fig. 7a) strongly adhered to fresh, ex vivo, human articular cartilage surfaces within 1 h (Fig. 7b) and remained attached for at least 3 days in continued culture. Attachment strength was qualitatively assessed using forceps to lift the combined construct by holding only the corner of the cell sheet. No peeling or detachment was observed during the forceps manipulation. Safranin-O staining after 3-days of co-culture showed close physical adhesion between the sheet and the cartilage surface, with few to no gaps seen along the interface (Fig. 7c). Laminin staining after 3-days of co-culture was most intense at the interface between the sheet and the cartilage surface (Fig. 7d), supporting continued adhesion through possible biological binding between cell sheets and target cartilage tissue.

## Discussion

Many studies demonstrate that MSCs exhibit increased chondrogenic potential in 3D structures compared to 2D structures^22,60–62^. Unlike 2D cell cultures, 3D cultures allow cells to assume rounded cell morphologies associated with chondrocyte cytoskeletal organization^26,51^. The contracted cell sheets spontaneously produce this 3D environment for cells to assume a more rounded and less elongated cytoskeletal structure, which is directly related to their chondrogenic potential^63, 64^. The cytoskeletal reorganization and transition from 2 to 3D culture seen in contracted cell sheets upon temperature-mediated detachment (Figs. 2, 3) are most likely caused by changes in stromal cell tensegrity, where cell release from anchored/adherent culture allows spontaneous contraction of actin filaments, prompting cell contraction within cell sheets^65^.

Cell sheet technology spontaneously detaches cells as confluent sheets without harvesting enzymes or damage to the endogenous cell–cell and cell-ECM interactions, maintaining endogenous cellular contractile forces of these collective interactions along actin filaments, which stimulates sheet contraction as a contiguous unit^40,66,67^. This post-detachment cell sheet contraction spontaneously fabricates 3D, multi-nuclei thick, scaffold-free cell sheet structures (Fig. 2) and induces cytoskeletal reorganization (Fig. 3). These changes in cytoskeletal structure may also stimulate mechanotransduction^68,69^, mimicking early chondrogenic condensation by changing both cell shape and ECM structure, resulting in increases in cell–cell interactions via β-catenin and upregulation of pro-chondrogenic signaling molecules BMP2 (regulator of cellular condensation)^70^ and COMP (regulator of collagen accumulation and ECM assembly)^71^ prior to chondrogenic induction.

Structural transitions and upregulation of pro-chondrogenic signaling prior to chondrogenic induction results in 3D contracted cell sheets achieving chondrogenic phenotypes after induction with chondrogenic medium (Fig. 4). Healthy articular cartilage specifically has hyaline chondrogenic phenotypes, including ECM rich in type II collagen and proteoglycans (which enable resistance to shear, compressive, and tensile forces), expression of SOX9 and aggrecan, low expression of type I collagen, nuclei in lacunae structures, and low cellular densities relative to ECM^4,72^. The harvested 3D contracted sheets successfully achieve these standard and accepted benchmarks of hyaline-like phenotypes after differentiation: significant type II collagen and proteoglycan content in the ECM, high expression of common hyaline cartilage markers (SOX9, COL2A1, ACAN), low expression of type I collagen with a high COL2A1/COL1A1 ratio, and rounded cell structures with nuclei in lacunae structures at relatively low cellular densities. In addition to ECM composition (i.e. proteoglycan, aggrecan, and type II collagen content), ECM deposition (as seen in differentiating 3D contracted sheets) is also associated with hyaline chondrogenic differentiation^14^. The cytoskeletal reorganization within 3D contracted cell sheets prior to chondrogenic induction upregulates COMP and BMP2, which are directly associated with ECM assembly and collagen accumulation^71,73^, resulting in significantly more ECM deposition in the 3D cell sheets than in the 2D cultures after chondrogenic induction. 3D cell sheets, generated from spontaneous contraction upon temperature-mediated detachment from culture surfaces, are initially cell-dense structures; however, ECM deposition that significantly increases 3D cell sheet thickness during chondrogenesis decreases the construct’s overall cellular density. This reduction in overall cellular density from ECM deposition results in a hyaline-like tissue construct that more closely matches native hyaline cartilage structure and cellular distribution^4^.

A major limitation of MSC chondrogenic differentiation to hyaline-like phenotypes is the inevitable progression of MSC-derived chondrocytes towards hypertrophy and fibrocartilage both in vitro and in vivo^74^. Although 3D structures are clearly necessary for proper hyaline-like chondrogenic differentiation, specific thresholds must be determined as construct thickness and cellular densities have been shown to impact media diffusion, affecting chondrogenic differentiation and hypertrophy by creating areas of low oxygen tension and increasing nutrient diffusion gradients in thicker tissues^20,75^. In this study, 3D cell sheets exhibited a similar progression of chondrogenic development, but a delayed onset of hypertrophic characteristics compared to standard positive control 3D pellet cultures in vitro (Fig. 5). Although the driving mechanism of MSC-derived chondrocyte hypertrophy in vitro is still largely unknown^74,76^, the observed delayed onset of MSC sheets’ hypertrophic characteristics in the present study most likely results from the sheets’ structural characteristics. Specifically, 3D cell sheets used in this study are thinner, with reduced cellular densities, compared to control 3D pellet cultures, promoting more substantial and uniform media diffusion throughout the construct, allowing chondrogenesis to be primarily driven by media supplementation rather than cellular signaling. Continued over-expression of cellular signaling, such as β-catenin, during chondrogenic differentiation has been linked to inducing chondrocyte hypertrophy in vitro via activation of the canonical Wnt pathway^57–59^. The 3D pellet cultures’ greater thickness, combined with higher cellular densities, present a barrier to sufficient nutrient diffusion, which may increase reliance on these cell–cell interactions [i.e. β-catenin upregulation (Fig. 5)] to propagate chondrogenic cues to cells at the pellets’ cores poorly exposed to free media. These data together suggest that tailoring construct thickness and cellular densities of 3D cell sheets may modulate cellular interactions during chondrogenesis, delaying the onset of MSC-derived chondrocyte hypertrophy in vitro. It is important to assess and monitor any transitions toward hypertrophic or fibrocartilage phenotypes during in vitro differentiation as these phenotypes would be detrimental to enacting prolonged therapeutic benefit in future in vivo studies requiring stable hyaline-like tissue. Further in vitro studies are necessary to elucidate optimal construct thicknesses and specific mechanisms driving this hypertrophic transition and identify the most effective sheet preparation parameters for producing hyaline cartilage in vitro while preventing MSC-derived chondrocyte hypertrophy.

Various differentiation platforms have successfully promoted chondrogenic differentiation of MSCs to hyaline-like phenotypes in vitro; however, none of these differentiation products has been successfully translated to human applications^9,11^. For example, pellet cultures are used primarily for in vitro verification of differentiation potential rather than in vivo therapeutic applications, based on limitations in adhesion capabilities and homogeneity of regenerated tissue^27,28^. Clusters of pellet cultures have been used to fill cartilage defects in vivo, and have shown some capacity to populate the negative space left by the pellets’ spherical shape constraints^27,28^. However, these pellet clusters do not create homogenous tissues and do not strongly adhere to biologic surfaces without additional glues or support membranes to contain them at defect sites^27,28^.

One unique benefit of cell sheet technology is the ability to directly and spontaneously transplant cells without scaffolds or support materials to target tissue sites via retention of endogenous ECM, cell interactions, and intact adhesion proteins, which also provide a stable cell culture environment for interacting with the native tissues^40,41,44,66^. Our data show that cell sheets can be transferred after differentiation, adhere to biologic surfaces, and that this transfer does not affect the cell sheets’ characteristics (Fig. 6). Maintenance of sheet chondrogenic and structural characteristics, in terms of sulfated proteoglycan staining and gross morphological structure, after manipulation and transfer is promising for rapidly replacing damaged or missing hyaline cartilage in future in vivo therapeutic applications. These differentiated cell sheets were also able to adhere strongly to fresh ex vivo human cartilage and potentially begin interfacing with native chondrocytes within 3 days (Fig. 6). These endogenous adhesion capabilities are attributed to retention of adhesion molecules along the sheets’ basal surface post-differentiation, which are not abundant along the periphery of the pellet cultures, likely causing the disparity in adhesion capabilities. Retention of surface adhesion molecules is expected to aid in cell sheet engraftment and localization at cartilage defect sites. Close physical and biochemical interfacing between hyaline-like cell sheets and native cartilage is expected to help maintain sheets’ hyaline characteristics in vivo via direct chondrogenic signaling from the host cartilage.

This ex vivo experiment represents an ideal interaction between the cell sheets and healthy articular cartilage in the absence of all other defect microenvironmental or mechanical factors that would influence transplant adhesion and survival in vivo. Additionally, this model places the cell sheets in contact with healthy superficial layers of cartilage, rather than deeper calcified cartilage, which would generally be the interface in pre-clinical and clinical applications. Previously, chondrocyte sheets have been shown to successfully adhere to healthy superficial layers of cartilage in vitro^77^, as well as adhere and survive in vivo within the knee environment through spontaneous attachment to calcified cartilage and subchondral bone through pre-clinical and small cohort clinical focal chondral defect studies^46,48,49,78,79^. Based on this precedent, our attachment study aimed to first assess if post-differentiation MSC sheets retain this established cell sheet adhesion ability with healthy superficial cartilage, in addition to standard culture-plastic dishes, before moving forward with more complex transplantation studies that will fully recapitulate the cartilage defect environment. To fully verify sheet transplantation capabilities and therapeutic benefits for these chondrogenically differentiated cell sheets in articular cartilage focal defects, in vivo testing with long-term follow-ups and specific focus on cell sheet fate and xenogeneic immune response will be necessary.

In this study, we demonstrate that (1) MSC sheets are able to chondrogenically differentiate to hyaline cartilage in vitro without scaffold materials after spontaneous post-detachment cell sheet contraction via structural transformation and cytoskeletal reorganization, (2) these 3D MSC sheets provide a suitable initial thickness and cellular density that delays hypertrophy while maintaining hyaline-like chondrogenic phenotypes in vitro, and (3) after differentiation, these 3D cell sheets can be manipulated without damaging the chondrogenic construct and spontaneously adhere directly to cartilage surfaces, potentially interfacing with the native tissue via retained adhesion proteins. Based on these findings, we assert that 3D MSC sheets represent a distinct platform for further developing scaffold-free hyaline cartilage constructs for future transplantable articular cartilage regeneration therapies.

## Conclusions

Cell sheet-based technology presented in this study represents an improved strategy for fabricating scaffold-free cartilage constructs with hyaline-like characteristics in vitro. Furthermore, hyaline-like chondrogenically differentiated 3D MSC sheets spontaneously adhere to cartilage tissue without damaging key cell sheet characteristics. Our cell sheet-based technique using MSCs should provide an adaptable platform to generate hyaline-like constructs in vitro for future applications that rapidly and directly replace damaged hyaline articular cartilage in vivo.

## Materials and methods

### Cell culture

hBMSCs were purchased from Lonza (donor: 33 Y, male) at Passage 2. These cells were positive for CD44, CD105, and CD90, negative for CD45, CD19, and HLA-DR, and express multipotent capabilities (COA, Lot#0000684888). hBMSCs were plated at 3000 cells/cm^2^ in growth media containing High-Glucose (4.5 g/L) Dulbecco’s Modified Eagle’s Medium (HG-DMEM) (Life Technologies, CA, USA) supplemented with 10% fetal bovine serum (FBS) (Thermo Fisher Scientific, MA, USA), 1% penicillin streptomycin (PS) (Gibco, NY, USA), and 5 ng/mL basic fibroblast growth factor (bFGF) (PeproTech, NJ, USA) and incubated in a humidified environment (37 °C, 5% CO_2_). Media was changed at day 1 and day 5, and cells were cultured until 90% confluent, approximately 7 days. Cells were passaged using 0.05% Trypsin–EDTA (Gibco) and the cell suspensions were counted using a hemocytometer. Cells were expanded and banked at Passage 3 and 4 and used for experimentation at Passage 5.

### Cell sheet and pellet fabrication and chondrogenic differentiation

Passage 5 hBMSCs were aliquoted in 20% FBS growth media^80^ at 2.5 × 10^5^ cells in 15 mL conical tubes for standard pellet cultures^22,61^, and seeded at 6.7 × 10^4 ^cells/cm^2^ into 1.0 µm-diameter pore, 6-well cell culture inserts (Falcon, NE, USA) for monolayer cultures and into 35 mm diameter UpCell dishes (CellSeed, Tokyo, Japan) for cell sheet cultures based on previous methods for cell sheet fabrication^81,82^. For pellet fabrication, conical tubes were centrifuged at 500 × *g* for 10 min. Caps were loosened and cells were transferred to a standard incubator (37 °C, 5% CO_2_) for 3 days to allow for pellet formation. For cell sheet fabrication, cells were cultured for 5 days to reach confluence. At 5 days, cell sheets were moved to 20 °C for 1 h, then detached with forceps. For re-plating cell sheets, 1.0 µm-diameter pore, 6-well cell culture inserts were conditioned with FBS overnight prior to re-plating the cell sheets to aid in adhesion. Inserts were washed twice with 1 × phosphate buffered saline (PBS) (Gibco) to remove residual FBS before sheet transfer. Detached cell sheets were transferred to the conditioned cell culture inserts using overhead projector polyester film (Apollo, NY, USA) to ensure basal contact with insert well culture surfaces and incubated in 20 µL growth media in a standard incubator for 1 h. After 1 h, fresh cell growth media was added to the sheets and they were incubated for an additional 3 days to ensure sheet attachment and mirror pellet culture incubation periods. After the 3-day incubation step, chondrogenic samples were induced with chondrogenic medium, control samples were kept in 10% FBS cell growth media, and all samples were transferred to a hypoxia incubator (37 °C, 5% CO_2_, 5% O_2_). Chondrogenic medium contained HG-DMEM supplemented with 10 ng/mL transforming growth factor beta-3 (TGFβ3) (Thermo Fisher Scientific), 200 ng/mL bone morphogenic protein-6 (BMP6) (PeproTech), 1% Insulin-Transferrin-Selenium (ITS-G) (Thermo Fisher Scientific), 1% PS (Life Technologies), 1% non-essential amino acids (NEAA) (Thermo Fisher Scientific), 100 nM dexamethasone (MP Biomedicals, OH, USA), 1.25 mg/mL bovine serum albumin (BSA) (Sigma-Aldrich, MO, USA), 50 µg/mL L-ascorbic acid 2-phosphate (Sigma-Aldrich), 40 µg/mL L-proline (Sigma-Aldrich), and 5.35 µg/mL linoleic acid (Sigma-Aldrich). Media composition was based on previously reported components and concentration ranges^83^. For chondrogenic and control samples, media was changed twice a week for the duration of differentiation (day 0–3 weeks).

### Histological analysis

After fixation with 4% paraformaldehyde (PFA) (Thermo Scientific) for 15 min, samples were paraffin embedded. Embedded samples were sectioned at 4 µm. To identify cell morphology, Hematoxylin and Eosin (H&E) staining was conducted according to standard methods^84^. Briefly, samples were stained for 4 min with Mayer’s Hematoxylin (Sigma-Aldrich) and 4 min with Eosin (Thermo Scientific). To detect mature chondrogenesis, Safranin-O staining was conducted according to standard methods^84^. Briefly, samples were stained for 4 min with Wiegert’s Iron Hematoxylin (Sigma-Aldrich), 5 min with 0.5 g/L Fast green (Sigma-Aldrich), and 8 min with 0.1% Safranin-O (Sigma-Aldrich). All samples were dried overnight before being imaged with a BX 41 widefield microscope (Olympus, Japan) using AmScope Software (v4.8.15934, USA). Safranin-O stained slide cross sections were used to calculate cell sheet thicknesses and nuclei densities. For each cell sheet slide, 3 pictures were taken along the length of the cell sheet. Using the measurement tools built into the AmScope software, 5 measurements from the apical to basal edge of the sheet were made per picture, and these measurements were evenly spaced out along the sheet. Nuclei counting was done using the same 3 pictures/sheet. Using the measurement tools built into the AmScope software, a 500 µm length of the cell sheet was marked. The number of nuclei were counted within the marked section using ImageJ software (v.1.51, NIH, USA). For cell sheet diameter calculations, macroscopic images of the sheets were analyzed using ImageJ software. Five diameter measurements were made for four cell sheets per group. All measurements were averaged for each sample group.

### Immunohistochemical analysis

For cross-sectional IHC analysis, samples were fixed on the insert membrane with 4% PFA for 15 min and paraffin embedded. Embedded samples were sectioned at 4 µm and stained for type II and type I collagen to detect mature chondrogenesis, MMP13 to detect hypertrophy, and laminin to detect adhesive molecules. Briefly, antigen retrieval was conducted by incubating with a 1:50 dilution of 50 × Low pH Target Retrieval Solution (Dako, Agilent Technologies, CA, USA) for 15 min at 106–110 °C at low pressure in a pressure cooker for type II collagen samples and with Proteinase K (Dako, Agilent Technologies) for 6 min at room temperature (RT) for type I collagen, MMP13 and laminin samples. Laminin samples were permeabilized with 0.1% Triton X-100 (Sigma-Aldrich) for 10 min at RT. Non-specific binding was blocked for type II collagen, MMP13, and laminin samples with 10% Normal Goat Serum (Vector Laboratories, CA, USA) and with 5% Normal Donkey Serum (Abcam) for type I collagen samples, at RT for 30 min. Type II collagen, type I collagen, MMP13, and laminin samples were then incubated in a 1:200 dilution of Anti-Collagen Type II primary antibody (Thermo Fisher Scientific), a 1:200 dilution of Anti-Collagen Type I primary antibody (Thermo Fisher Scientific), a 1:200 dilution of Anti-MMP13 primary antibody (Abcam), or a 1:100 dilution of Anti-laminin primary antibody (Abcam) at 4 °C overnight, respectively. Samples were washed with 1 × PBS and incubated in either a 1:200 dilution of Goat Anti-Rabbit 488 secondary antibody (Thermo Fisher Scientific) for type II collagen, MMP13, and laminin samples or a 1:200 dilution of Goat Anti-Donkey 594 secondary antibody (Thermo Fisher Scientific) for type I collagen samples at RT, covered, for 2 h. After 2 h, samples were washed with 1 × PBS and mounted with a DAPI-containing mounting medium (Invitrogen, MA, USA). Samples were visualized with a Zeiss Axio widefield microscope and Zeiss ZEN software (v.2.7). To determine cytoskeletal arrangement, phalloidin (F-actin) staining was conducted. Briefly, samples were permeabilized with 0.1% Triton X-100 (Sigma-Aldrich) for 15 min and washed with 1 × PBS. Samples were then incubated with a 1:100 dilution of phalloidin AlexaFluor 488 (Life Technologies) at RT, covered, for 30 min. Samples were then washed with 1 × PBS and incubated with DAPI solution (2 drops/mL, Life Technologies) at RT for 5 min. Samples were then washed with 1 × PBS and prepared for mounting. Samples were imaged with a confocal microscope (Nikon A1 microscope, NIS Elements AR Software, v.4.30.01). Images were prepared using ImageJ software.

### Real-time quantitative PCR analysis

RNA from samples was extracted using 1 mL TRIzol/sample (Ambion, Life Technologies, CA, USA) with a pestle motor mixer. Total RNA was isolated with the PureLink RNA Mini Kit (Invitrogen, Thermo Fisher Scientific) according to manufacturer instructions. For cDNA synthesis, all comparative samples were synthesized at the same time. Before synthesizing cDNA, the RNA was quantified with a NanoDrop spectrophotometer (Thermo Scientific, USA), and all cDNA samples were prepared from 1 µg of RNA/sample. All samples with a purity (A_260_/A_280_) greater than 1.8 were deemed pure enough to use. cDNA synthesis was conducted using a High Capacity cDNA Reverse Transcriptase Kit (Applied Biosystems, Thermo Fisher Scientific, MA, USA) as per manufacturer instructions. Real-time qPCR analysis was conducted with TaqMan Universal PCR Master Mix (Applied Biosystems, Thermo Fisher Scientific) using an Applied Biosystems Step-OnePlus instrument. Gene expression levels were analyzed for the following genes: (1) glyceraldehyde 3-phosphate dehydrogenase (GAPDH, Hs99999905_m1) as a housekeeping gene, (2) β-actin (Hs99999903_m1), (3) β-catenin (Hs00355049_m1), (4) bone morphogenic protein 2 (BMP2, Hs00154192_m1), (5) cartilage oligomeric matrix protein (COMP, Hs00164359_m1), (6) SRY-box 9 (SOX9, Hs01001343_g1), (7) aggrecan (ACAN, Hs00153936_m1), (8) collagen type II alpha 1 chain (COL2A1, Hs00264051_m1), (9) collagen type I alpha 1 chain (COL1A1, Hs00164004_m1), (10) collagen type X alpha 1 chain (COLX, Hs00166657_m1), (11) matrix metallopeptidase 13 (MMP13, Hs00942584_m1), (12) Runt-related transcription factor 2 (RUNX2, Hs01047973_m1). All primers were manufactured by Applied Biosystems. Relative gene expression was calculated by the quantitative comparative CT method^85^. Gene expression was normalized to GAPDH expression levels. For cytoskeletal analysis and time comparison differentiation, expression levels are relative to the 0-day 2D monolayer control group, and for chondrogenic differentiation, expression levels are relative to the 2D monolayer 3-week control group.

### Post-differentiation manipulation and re-attachment

For assessing structural changes during post-differentiation manipulation and adhesion capabilities, 3-week chondrogenically differentiated contracted hBMSC sheets were cut in half using a scalpel. Half of each sheet was immediately fixed in 4% PFA for 15 min and paraffin embedded. The other half of each sheet was re-plated onto FBS-coated 35 mm tissue culture plastic dishes. To transfer the cell sheets, cell sheet halves were nudged off cell culture insert membranes and manually transferred to new FBS-coated culture dishes with forceps. After placement on the secondary surface, sheets were incubated in chondrogenic medium for 1 h to attach. After 1 h, fresh chondrogenic media was added and cell sheets were moved to a hypoxia incubator for further culture. Brightfield images were captured of cells at sheet edges between 0 and 72 h during the 3-day culture period with a Zeiss Axio widefield microscope and ZEN software. After 3 days, the cell sheet halves were fixed in 4% PFA for 15 min and paraffin embedded. For comparison, 3-week chondrogenically differentiated pellets were also re-plated onto FBS-coated 35 mm tissue culture plastic dishes. Pellets were manually transferred to the new culture surfaces by gentle pipetting. After placement on the secondary surface, pellets were incubated in a small amount of chondrogenic medium for 1 h to attach. After 1 h, fresh chondrogenic media was added and pellets were moved to a hypoxic incubator and cultured for an additional 6 h. Construct attachment efficacy was quantified as a ratio of attached sheets or pellets after this additional 6 h of culture to total number of sheets or pellets transferred (*n* = 6).

### Post-differentiation adhesion capabilities

To determine preliminary adhesion capabilities to healthy cartilage tissue, 3-week chondrogenically differentiated contracted sheets were cut in quarters using a scalpel. One quarter of each sheet was immediately fixed in 4% PFA for 15 min and paraffin embedded to act as a phenotypic and structural control. The other quarters of each sheet were transferred onto the apical side of fresh (same day as harvest) ex vivo human articular cartilage pieces (~ 2 cm^2^) harvested as de-identified tissue discards from human hip articular cartilage during routine hip arthroscopy procedures (donor: 25 Y, female). Cell sheets were allowed to adhere in a small amount of media for 45 min, after which adhesion was qualitatively checked with forceps and the construct was immersed in chondrogenic media and transferred to a hypoxia incubator. After 3 days co-culture in a hypoxia incubator, the cell sheet-on-cartilage samples were fixed in 4% PFA for 3 days and paraffin embedded.

### Statistical analysis

All statistical analysis was completed on data sets of *n* ≥ 4 biological replicates via at least 2 experimental repetitions and incorporating technical replicates to ensure consistency of results^85^. All quantitative values are expressed as a mean ± standard deviation. The D’Agostino-Pearson omnibus K2 test was used to determine normality of data sets. As such, a 2-tailed paired student’s *t*-test was used to evaluate significance in single variable comparison data sets (Figs. 2e,f, 3c–f, 4q–t), and a two-way analysis of variance (ANOVA) with Bonferroni testing was used to evaluate significance in multiple-variable data sets (Figs. 4o,p, 5s–u). Statistical significance was defined as not significant (ns) *p* ≥ 0.05, *p < 0.05, and **p < 0.01. All statistical analysis was conducted using GraphPad Prism software (v.6, https://www.graphpad.com/scientific-software/prism/).

## Supplementary information

Supplementary Figures.

## Data Availability

The data sets used or analyzed during the presented study are available from the corresponding authors upon reasonable request.
