# Supplementary material for: Fabrication of hyaline-like cartilage constructs using mesenchymal stem cell sheets
Source: Sci Rep. 2020 Nov 30;10:20869. doi: 10.1038/s41598-020-77842-0 (PMC7705723; doi:10.1038/s41598-020-77842-0)
Supplement: Supplementary file 1 — Supplementary Figures. [file 41598_2020_77842_MOESM1_ESM.docx]

Supplementary Information

**Fabrication of Hyaline-like Cartilage Constructs Using Mesenchymal Stem Cell Sheets**

*Hallie Thorp, Kyungsook Kim^*^, Makoto Kondo, David W. Grainger, Teruo Okano^*^*

1. **Materials and Methods**
   1. **Chondrogenic Potential of hBMSCs**

Differentiation method for chondrogenic hBMSC pellets is described in method section “Cell Sheet and Pellet Fabrication and Chondrogenic Differentiation” of the main manuscript. After fixation with 4% PFA (Thermo Scientific) for 15 min, samples were paraffin embedded. Embedded samples were sectioned at 4 μm. To detect early chondrogenesis, Alcian blue staining was conducted according to standard methods (10 min staining with Alcian blue solution (pH 2.5) (Sigma-Aldrich)). All samples were dried overnight before being imaged with a BX 41 widefield microscope (Olympus, Japan) using AmScope Software (USA).

1. **Results**

**2.1. Chondrogenic Potential of hBMSCs**

After 3 weeks in chondrogenic media and hypoxic conditions, hBMSCs as pellet cultures showed expected positive chondrogenic characteristics (Fig. S1). The 3-week differentiated pellets were positive for all histological chondrogenic stains (Alcian blue (Fig. S1d), Safranin-O (Fig. S1e), type II collagen (Fig. S1f)). The 3-week control pellets were negative for all chondrogenic stains (Fig. S1a-c). Gene analysis with quantitative real-time PCR showed significantly increased expression of chondrogenic markers (*SOX9* (Fig. S1g) (*p* = 0.0137), type II collagen (Fig. S1h) (*p* = 0.00135), aggrecan (Fig. S1i) (*p* = 0.00398)) for 3-week differentiated pellets compared to 3-week control pellets. These data reinforce the chondrogenic potential of the hBMSCs.


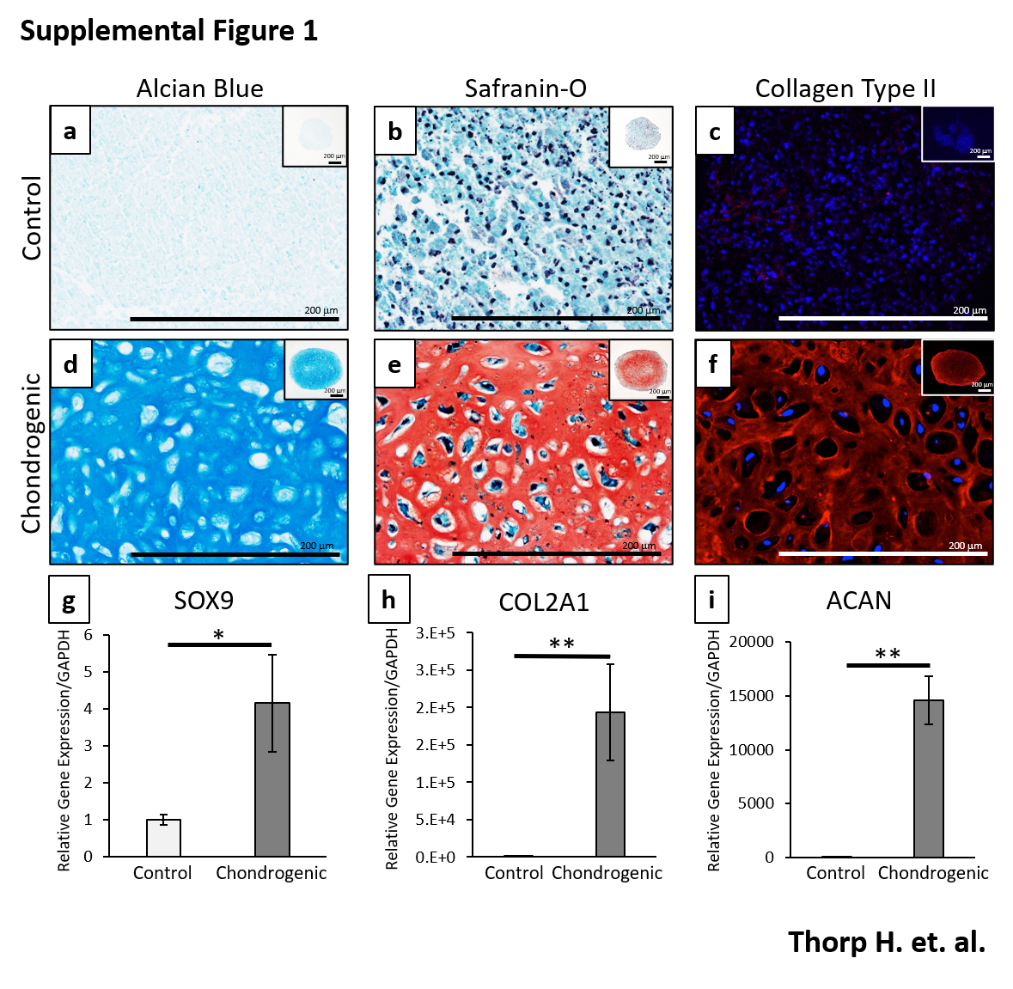


**Figure S1. Differentiation potential of hBMSCs in standard 3D pellet cultures.** Representative images of histological sections of pellets in (**a-c**) control medium and (**d-f**) chondrogenic medium after 3 weeks. Stains were (**a,d**) Alcian blue for acidic mucins, (**b,e**) Safranin-O/Fast green for sulfated proteoglycans, and (**c,f**) type II collagen (pseudo red) with DAPI (blue). (**g-i**) Chondrogenic gene expression with quantitative real-time PCR for (**g**) SOX9, (**h**) type II collagen, and (**i**) aggrecan. All gene expression normalized to GAPDH and compared to the control samples. Error bars represent means ± SD (*n* = 4) (**p*<0.05, ***p*<0.01).
